# The effects of short video app–guided loving-kindness meditation on interpersonal mindfulness, empathy, collaboration, affect, and workplace well-being among working professionals

**DOI:** 10.3389/fpubh.2025.1716315

**Published:** 2025-12-17

**Authors:** Chao Liu, Yehui Tu, Li-Jen Lin, Hao Chen, Thu-Hua Liu, Huang-Li Lin, Wen-Ko Chiou

**Affiliations:** 1 School of Journalism and Communication, Hua Qiao University, Xiamen, China; 2 General Education Center, Mindfulness Meditation Center, Ming Chi University of Technology, New Taipei, Taiwan; 3 Business Analytics Research Center, Chang Gung University, Taoyuan, Taiwan; 4 School of Film Television and Communication, Xiamen University of Technology, Xiamen, China; 5 Department of Industrial Design, Mindfulness Meditation Center, Ming Chi University of Technology, New Taipei, Taiwan; 6 Department of Psychiatry, Chang Gung Memorial Hospital, Taoyuan, Taiwan; 7 Department of Industrial Design, Chang Gung University, Taoyuan, Taiwan

**Keywords:** loving-kindness meditation, interpersonal mindfulness, empathy, interdisciplinary collaboration, positive and negative affect, workplace well-being, short video intervention

## Abstract

**Objective:**

This study examined the effectiveness of a short video app–guided Loving-Kindness Meditation (LKM) intervention in enhancing interpersonal mindfulness, empathy, interdisciplinary collaboration, affective states, and workplace well-being among working professionals.

**Methods:**

A randomized controlled trial (RCT) was conducted with 100 full-time employees (aged 25–60), randomly assigned to an intervention group (*n* = 50) or a control group (*n* = 50). The intervention group completed daily 3-min LKM sessions for 4 weeks via a secure short video app, while the control group received no intervention. Validated psychological scales were administered at baseline and post-intervention, including the Interpersonal Mindfulness Scale (IMS), Empathy Scale (ES), Interdisciplinary Collaboration Scale (ICS), Positive and Negative Affect Schedule–Short Form (PANAS-SF), and Workplace Well-being Scale (WWS). A 2 × 2 mixed ANOVA was used to assess group × time interaction effects.

**Results:**

Significant group × time interaction effects emerged for all six outcomes. Compared with controls, the intervention group showed greater increases in interpersonal mindfulness (*F* = 7.789, *p* = 0.006, η^2^ = 0.038), empathy (*F* = 9.831, *p* = 0.002, η^2^ = 0.048), collaboration (*F* = 4.832, *p* = 0.038, η^2^ = 0.022), and positive affect (*F* = 8.580, *p* = 0.004, η^2^ = 0.042), along with reduced negative affect (*F* = 10.169, *p* = 0.002, η^2^ = 0.049) and improved workplace well-being (*F* = 5.660, *p* = 0.018, η^2^ = 0.028).

**Conclusion:**

These findings demonstrate that short video–based LKM is a feasible and effective digital intervention for cultivating prosocial qualities, emotional regulation, and psychological well-being in workplace settings. The study supports integrating compassion-based practices into organizational wellness initiatives to promote employee functioning and workplace harmony.

**Clinical trial registration:**

https://www.chictr.org.cn/bin/project/edit?pid=285511, ChiCTR2500108223.

## 1 Introduction

In contemporary organizational environments, employees face escalating emotional, cognitive, and interpersonal demands. The fast-paced, digitalized nature of work—characterized by information overload, continuous multitasking, and blurred boundaries between professional and personal life—has contributed to growing levels of stress, emotional fatigue, and social disconnection, as demonstrated in recent occupational health studies by Sonnentag and Fritz (1). These challenges underscore an urgent need for psychological interventions that simultaneously strengthen emotional regulation, empathy, and interpersonal collaboration, thereby supporting both productivity and mental well-being in the workplace.

In recent years, mindfulness-based interventions have gained wide recognition for their effectiveness in cultivating self-awareness, attentional control, and emotion regulation in occupational contexts, as discussed by Hülsheger et al. (2) and Lomas et al. (3). Among these approaches, Loving-Kindness Meditation (LKM) represents a compassion-centered practice that extends feelings of goodwill toward oneself and others. Empirical research has shown that LKM enhances positive emotions, empathy, and prosocial behaviors by fostering warm affective states and reducing negative emotionality, as found in the work of Fredrickson et al. (4) and Kok et al. (5). Such mechanisms are particularly meaningful in workplaces, where empathy, compassion, and emotional balance play critical roles in teamwork, leadership, and conflict resolution, as noted by Donald et al. (6).

Mindfulness itself is commonly defined as a state of non-judgmental awareness that arises from deliberately paying attention to present-moment experiences, a concept introduced by Kabat-Zinn (7). The practice integrates two fundamental components: self-regulated attention, which sustains awareness of current experience, and an attitude of openness and acceptance toward that experience, as conceptualized by Bishop et al. (8). A substantial body of evidence indicates that mindfulness enhances emotional regulation, attentional stability, and resilience across a wide range of clinical and organizational contexts, according to Good et al. (9) and Goyal et al. (10).

Within this broader theoretical framework, LKM can be understood as a compassion-oriented extension of mindfulness. Whereas traditional mindfulness emphasizes awareness and acceptance, LKM actively cultivates benevolent emotions toward oneself, close others, and even difficult individuals, as highlighted in studies by Hofmann et al. (11) and Weng et al. (12). The affective warmth elicited through this practice aligns with the Broaden-and-Build Theory of Positive Emotions, proposed by Fredrickson (13), which posits that positive emotional states expand individuals’ thought–action repertoires and accumulate enduring psychological resources. In workplace contexts, such broadened states can enhance interpersonal mindfulness, prosocial motivation, and collaboration, as supported by the empirical findings of Donald et al. (6).

Despite its well-documented psychological benefits, the integration of LKM into organizational environments remains limited. Traditional meditation programs often require long sessions and in-person facilitation, which pose accessibility challenges for employees facing demanding schedules, as noted by Bartlett et al. (14). As modern work becomes increasingly digital and fragmented, there is a pressing need for scalable, technology-supported interventions that retain the efficacy of compassion-based practices while adapting to contemporary work rhythms.

Addressing this gap, the present study introduces a short video app–guided LKM intervention aimed at promoting compassion-based emotional regulation and social connectedness among working professionals. Unlike existing mobile mindfulness applications such as Headspace or Calm, which rely primarily on lengthy audio-based sessions requiring sustained attention, short video platforms employ micro-learning structures—brief, visually enriched, and emotionally engaging clips—that integrate naturally into users’ everyday media habits. This format not only lowers cognitive and temporal barriers to participation but also leverages algorithmic personalization and interactive feedback to sustain engagement over time.

Conceptually, this study bridges digital compassion training with media engagement theory, proposing that short video–guided LKM can activate comparable contemplative mechanisms while enhancing accessibility, adherence, and ecological validity in real-world professional settings. Practically, it contributes to workplace mental health research by offering a low-burden, scalable intervention model tailored to the realities of contemporary professionals who may lack the time or opportunity to participate in traditional mindfulness programs.

## 2 Literature review

### 2.1 LKM and interpersonal mindfulness

Loving-Kindness Meditation (LKM) is a contemplative practice rooted in cultivating unconditional kindness and compassion toward oneself and others. Unlike focused attention meditation, which centers on present-moment awareness, LKM emphasizes the intentional generation of prosocial emotional states and their extension outward across relational boundaries. This orientation has direct implications for interpersonal mindfulness, which involves awareness and non-reactivity in social contexts. Pratscher et al. (15) found that individuals who engage in LKM show greater attentiveness to their internal emotional states during interactions and report increased emotional regulation, empathy, and active listening. LKM may enhance interpersonal mindfulness by strengthening the practitioner’s capacity to notice emotional cues, suspend judgment, and respond with conscious compassion.

### 2.2 LKM and empathy

Empathy encompasses both affective resonance and cognitive perspective-taking—abilities essential to effective communication and conflict resolution in organizational contexts. LKM has consistently demonstrated efficacy in increasing empathy by dissolving emotional barriers and enhancing one’s sense of shared humanity (11). Functional neuroimaging studies have shown that LKM activates areas of the brain associated with social cognition, such as the temporoparietal junction and medial prefrontal cortex (16). Moreover, LKM practices that guide participants to extend compassionate thoughts to others—ranging from loved ones to difficult individuals—train the mind to adopt multiple social perspectives, thereby increasing empathic accuracy and concern. This makes LKM especially relevant in diverse, interpersonal workplace settings where empathy is a core interpersonal skill.

### 2.3 LKM and collaboration

Effective collaboration depends not only on cognitive coordination but also on emotional openness, trust, and mutual respect. LKM supports these dimensions by reducing defensiveness, increasing affiliative motivation, and promoting inclusive attitudes (12). Compassion-focused practices like LKM have been associated with increased cooperation in economic games, greater willingness to help others, and more constructive group engagement (17). In workplace teams, where success often hinges on the ability to navigate diverse perspectives and resolve conflict, the emotional softening and openness fostered by LKM may translate into greater collaborative effectiveness and decreased interpersonal friction.

### 2.4 LKM and affect

Affective balance—defined by high levels of positive affect and low levels of negative affect—is a well-established outcome of LKM. The Broaden-and-Build Theory (13) suggests that positive emotions broaden individuals’ thought-action repertoires, enhancing resilience and flexibility. LKM actively cultivates emotions such as joy, gratitude, serenity, and love, which over time can build durable personal and social resources (4). Meta-analytic reviews have shown that LKM significantly reduces symptoms of depression, anxiety, and anger while enhancing subjective well-being (18). Compared to other forms of meditation, LKM is particularly effective in increasing emotional warmth and reducing harsh self-judgment and social negativity, thereby improving the overall emotional climate in workplace settings.

### 2.5 LKM and workplace well-being

Workplace well-being refers to employees’ holistic experience of emotional, psychological, and relational satisfaction in the context of work. It includes not only momentary mood states but also longer-term experiences of meaning, fulfillment, and connectedness (19). LKM can contribute to this by reducing internal stress, improving interpersonal harmony, and reframing one’s relationship to work through the lens of compassion and purpose. Practicing LKM has been associated with lower levels of burnout, higher job satisfaction, and stronger organizational citizenship behaviors (20). Its emphasis on kindness toward both self and others may buffer against workplace cynicism and increase employees’ emotional commitment to their roles.

Taken together, the reviewed literature demonstrates that LKM exerts multifaceted effects across emotional, cognitive, and interpersonal domains in occupational settings. These outcomes—enhanced interpersonal mindfulness, empathy, collaboration, affect regulation, and workplace well-being—share a common psychological mechanism: the cultivation of prosocial emotions that broaden awareness and strengthen relational resources. According to the Broaden-and-Build Theory of Positive Emotions (13) and Interpersonal Mindfulness Theory (15), compassion-based practices expand individuals’ attentional and cognitive repertoires, enabling them to respond to social interactions with greater empathy, flexibility, and awareness. In organizational contexts, such broadened emotional and cognitive states promote constructive communication, cooperative engagement, and sustained well-being. Building on this integrated framework, the present study proposes and empirically tests the following hypotheses.

### 2.6 Hypotheses

*H1*: Short video app–guided loving-kindness meditation will improve interpersonal mindfulness among working professionals.

*H2*: Short video app–guided loving-kindness meditation will enhance empathy, including both affective and cognitive dimensions.

*H3*: Short video app–guided loving-kindness meditation will increase employees’ willingness and ability to engage in interdisciplinary collaboration.

*H4*: Short video app–guided Loving-Kindness Meditation will improve affective balance by increasing positive affect and reducing negative affect in the workplace.

*H5*: Short video app–guided loving-kindness meditation will improve workplace well-being among full-time employees.

## 3 Methods

### 3.1 Study design

This study adopted a randomized controlled trial (RCT) design to evaluate the effects of a short video app–guided Loving-Kindness Meditation (LKM) intervention on working professionals’ interpersonal mindfulness, empathy, collaboration, affect, and workplace well-being. The intervention lasted for 4 weeks and included two assessment time points: baseline (T1) and post-intervention (T2). Participants in the intervention group received daily LKM content through a secure mobile short video app, while the control group received no intervention during the same period. All participants completed standardized questionnaires at both time points, and changes in the outcome variables were statistically analyzed using repeated measures.

### 3.2 Participants

Participants were full-time working professionals recruited from various industries across China through online platforms, corporate outreach, and professional networking channels. Inclusion criteria required participants to (1) be aged between 25 and 60, (2) hold a full-time job with regular digital media usage, and (3) report daily engagement with short video platforms such as Douyin or TikTok. Participants with prior experience in formal meditation, ongoing psychological treatment, or a history of psychiatric disorders were excluded to control for external influences on the intervention effect.

A total of 110 individuals completed the baseline assessment and met all inclusion criteria. After screening and informed consent procedures, 100 participants were randomly assigned to either the LKM intervention group (*n* = 50) or the control group (*n* = 50) using stratified randomization based on age and gender. The final sample had a mean age of 38.4 years (SD = 7.2), with the majority aged between 30 and 45. Gender distribution was 58% female (*n* = 58) and 42% male (*n* = 42), representing a diverse cross-section of white-collar professionals.

All participants reported daily use of short-form video apps, owned smartphones, and had no prior experience with LKM. The demographic characteristics of the sample are summarized in Table 1.

**Table 1:** Demographic characteristics of participants.

Variable	Intervention group (*n* = 50)	Control group (*n* = 50)
Age (years)	*M* = 38.6, SD = 7.1	*M* = 38.2, SD = 7.3
Gender	Male = 21, Female = 29	Male = 21, Female = 29
Occupation	Full-time professionals	Full-time professionals
Daily use of short video apps	100%	100%
Smartphone ownership	100%	100%
Prior meditation experience	None	None
Psychiatric history	None	None

### 3.3 Intervention description

Participants in the intervention group engaged in a structured four-week Loving-Kindness Meditation (LKM) program, delivered via a secure, password-protected mobile short video app compatible with both iOS and Android devices. Each participant received a unique login ID to ensure individualized tracking and data security. The short video contents were co-developed by two certified mindfulness instructors and one clinical psychologist from the Mindfulness Meditation Center at Ming Chi University of Technology, Taiwan. All video materials were expert-reviewed and pilot-tested to ensure consistency in language, pacing, and emotional tone.

The short video app–guided LKM program comprised four modules, each delivered through concise 1–2 min video segments. The modules included (1) self-compassion, (2) compassion for others, (3) expanding kindness toward difficult individuals, and (4) integration and reflection. Each video combined guided meditation with visual and auditory stimuli to foster affective engagement. Participants were encouraged to watch one video per day and engage in a brief reflection on their emotional state after each session.

Each session lasted approximately 3 min and featured professionally recorded guided instructions, integrating compassion-focused phrases, gentle breathing guidance, and soothing background music. The videos were visually minimalist, showing a neutral color palette, soft ambient lighting, and slow-motion nature visuals to reinforce calm and focus. The narrator adopted a warm, gender-neutral, and non-directive tone to encourage participants’ emotional receptivity and internal visualization.

The program was designed as a progressive curriculum, gradually expanding the scope of compassion targets across 4 weeks:

*Week 1*: Cultivating compassion toward oneself, using phrases such as “May I be safe, may I be happy.”

*Week 2*: Extending compassion toward close colleagues and team members within the participant’s work environment.

*Week 3*: Directing compassion toward neutral individuals in the broader organization or professional community.

*Week 4*: Offering compassion to difficult individuals or those with whom the participant had experienced tension or conflict.

Each video followed a three-part structure:

1. Opening (≈30 s): a short introduction contextualizing the day’s compassion focus;
2. Core meditation (≈2 min): guided repetition of compassion phrases combined with mindful breathing and imagery;
3. Closing reflection (≈30 s): a gentle visualization prompt encouraging awareness of emotional and interpersonal changes.

To support adherence, the app sent automated daily reminders at a self-selected time. These non-intrusive notifications encouraged participants to maintain a consistent meditation habit. The app also automatically recorded engagement data, including completion rate, session duration, and login frequency—providing an objective measure of adherence. Weekly summary emails reinforced the week’s compassion theme, offered brief psychological explanations, and encouraged continued integration of meditation into daily routines.

In terms of adherence and fidelity, participants demonstrated a high rate of engagement with the short video–guided LKM program, completing on average 93.5% (SD = 4.7%) of the assigned sessions. Fidelity to the intervention protocol was ensured through two mechanisms: (1) expert validation of all video scripts and voice recordings prior to release, ensuring conceptual and procedural alignment with LKM principles; and (2) automated in-app tracking that prevented fast-forwarding or skipping, thereby guaranteeing full exposure to each session. Weekly self-reports were cross-checked with system logs to confirm data accuracy and participant engagement quality.

**Figure 1:**
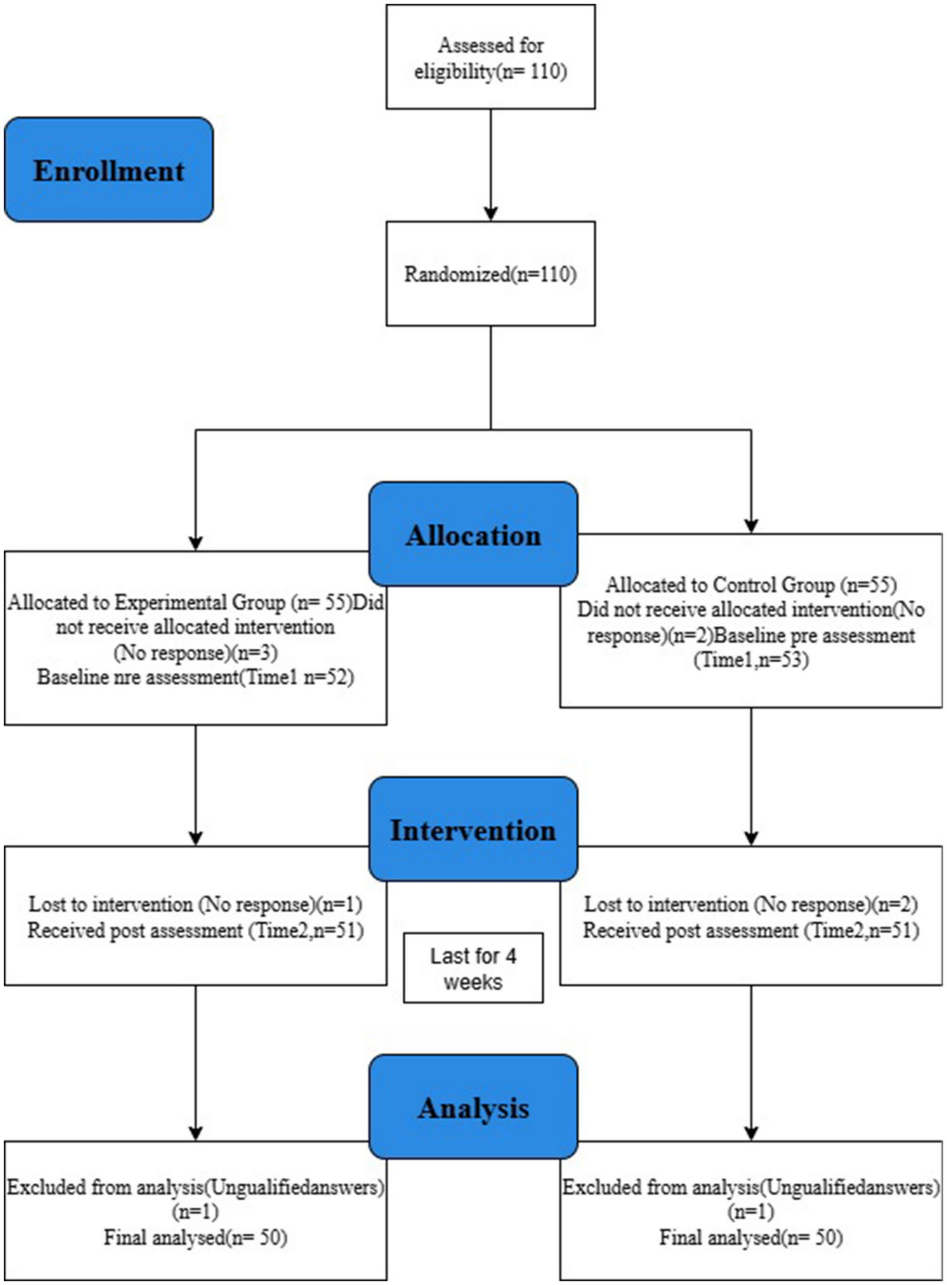
Procedure flow chart.

At the conclusion of the four-week intervention, participants completed post-intervention questionnaires and provided short written reflections describing emotional changes and interpersonal experiences. These qualitative reflections, combined with quantitative outcome measures, provided a multidimensional understanding of the intervention’s psychological effects. Participants were randomly assigned to either the intervention or control group using a computer-generated randomization sequence in SPSS Version 26.0. Group assignments were placed in sealed opaque envelopes by an independent researcher not involved in participant recruitment or assessment, ensuring allocation concealment. Participant flow through each stage of the trial—including enrollment, randomization, intervention, and analysis—is presented in Figure 1.

Participants assigned to the control group received no intervention during the study period and continued their normal work and lifestyle routines. To ensure ethical parity, control group participants were granted full access to all LKM video materials after the study ended.

### 3.4 Measures

#### 3.4.1 Interpersonal mindfulness

Interpersonal mindfulness was measured using the Interpersonal Mindfulness Scale (IMS) developed by Pratscher et al. (15). The IMS consists of 21 items that assess individuals’ awareness and attentional focus during social interactions, capturing three key dimensions: awareness of emotional reactions, attentiveness to thoughts, and awareness of bodily sensations. Participants rated each item on a 5-point Likert scale ranging from 1 (strongly disagree) to 5 (strongly agree), with higher scores indicating greater mindfulness in interpersonal contexts. Sample items include “I am aware of my emotional reactions when interacting with others” and “I pay attention to my thoughts when engaging with others.” The IMS has demonstrated robust psychometric properties and is grounded in contemporary mindfulness theory applied to interpersonal communication.

#### 3.4.2 Empathy

Empathy was measured using an adapted Empathy Scale (ES) (21) derived from design-thinking and human-centered practice contexts. The scale comprises 4 items that assess participants’ affective immersion and cognitive perspective-taking when solving problems, emphasizing understanding and situational engagement with stakeholders (e.g., users or individuals affected by the issue). Each item was rated on a 5-point Likert scale ranging from 1 (strongly disagree) to 5 (strongly agree), with higher scores indicating greater empathy in problem-solving contexts. Sample items include “I put myself in the position of the person concerned to understand their difficulties and needs” and “I adopt the perspective of the person concerned when thinking about a problem.” This scale is particularly suited to evaluating empathy-related thinking and action in workplace project implementation.

#### 3.4.3 Interdisciplinary collaboration

Interdisciplinary Collaboration Scale (ICS) was assessed using a 4-item scale (22) adapted from practice-oriented contexts, focusing on participants’ attitudes and abilities in cross-disciplinary teamwork during problem-solving and project execution. The scale evaluates four core dimensions: openness to cooperation, willingness to engage across fields, effective communication, and receptiveness to diverse perspectives. Each item was rated on a 5-point Likert scale ranging from 1 (strongly disagree) to 5 (strongly agree), with higher scores reflecting stronger collaborative tendencies. Sample items include “I believe teamwork is more effective than working alone in solving problems” and “I can communicate effectively with people from different professional backgrounds.” This instrument is particularly suited for workplace environments requiring integration of diverse knowledge and skills.

#### 3.4.4 Affect

Affect was measured using the short-form version of the Positive and Negative Affect Schedule (PANAS-SF) developed by Watson et al. (23). The PANAS-SF is a widely used instrument in psychological and health research for assessing transient emotional states. To enhance feasibility and reduce participant burden, this study adopted the 10-item short form, which includes 5 items each for positive affect (e.g., alert, inspired, determined, attentive, active) and negative affect (e.g., upset, hostile, ashamed, nervous, afraid). Participants rated the extent to which they were experiencing each emotion at the present moment on a 5-point Likert scale ranging from 1 (very slightly or not at all) to 5 (extremely). Higher scores in each subscale indicated greater intensity of positive or negative affective states.

#### 3.4.5 Workplace well-being

Workplace Well-being Scale (WWS) was assessed using a 6-item scale adapted from previous empirical studies on employee psychological functioning in organizational settings (24). The scale measures core dimensions of work-related well-being, including job satisfaction, engagement, sense of accomplishment, and perceived meaning at work. Each item was rated on a 5-point Likert scale ranging from 1 (strongly disagree) to 5 (strongly agree), with higher scores indicating a greater sense of well-being at work. Participants responded based on their current subjective experience in the workplace. This measure served as a key indicator for evaluating whether the digital intervention effectively enhanced employees’ positive psychological states within their occupational environment.

To ensure internal consistency within the present sample, Cronbach’s α coefficients were computed for each scale. The Interpersonal Mindfulness Scale (IMS) demonstrated excellent reliability (α = 0.92). The Empathy Scale (ES) showed good internal consistency (α = 0.86), and the Interdisciplinary Collaboration Scale (ICS) yielded a similarly high reliability (α = 0.88). For affective measures, the Positive Affect (PA) and Negative Affect (NA) subscales of the PANAS-SF exhibited strong reliability (α = 0.90 and α = 0.87, respectively). Finally, the Workplace Well-being Scale (WWS) achieved excellent internal consistency (α = 0.91). These values confirm that all instruments demonstrated satisfactory psychometric reliability in this occupational sample.

For clarity and consistency, the following abbreviations are used throughout the manuscript: IMS refers to the Interpersonal Mindfulness Scale, which measures mindful awareness and presence during interpersonal interactions; ES denotes the Empathy Scale, assessing both affective and cognitive dimensions of empathy; ICS stands for the Interdisciplinary Collaboration Scale, evaluating employees’ willingness and ability to collaborate across professional domains; WWS represents the Workplace Well-being Scale, capturing employees’ perceived satisfaction, engagement, and emotional balance at work; PA indicates Positive Affect, representing pleasant and activating emotional states; and NA indicates Negative Affect, reflecting distressing or deactivating emotional states. All abbreviations are defined at first mention and used consistently in the text, tables, and figures thereafter.

All instruments originated from validated English-language scales published in peer-reviewed journals. Since participants were Chinese-speaking managers, we employed a rigorous two-way translation process to ensure conceptual and linguistic accuracy. A bilingual Ph. D. researcher first translated the scales into Chinese. An English-speaking master’s student independently translated them back into English. This back-translation process followed Brislin’s (25) guidelines to maintain semantic equivalence.

Three bilingual experts in health psychology and communication reviewed both language versions for clarity and cultural relevance. We resolved discrepancies through discussion. Before the main study, we conducted a pilot test with 30 Chinese mid-level managers to assess item clarity and cultural appropriateness. Based on participant feedback, we made minor revisions to enhance readability.

### 3.5 Procedure

After completing baseline assessments (T1), participants were randomly assigned to either the intervention or control group using a computer-generated randomization list. Participants in the intervention group accessed daily Loving-Kindness Meditation (LKM) videos through the secure mobile application for 4 consecutive weeks, while control group participants continued with their usual routines without receiving any intervention during the study period. All participants completed follow-up measures at the end of week four (T2) to assess post-intervention outcomes. The flow of recruitment, randomization, and assessment procedures is depicted in Figure 1.

### 3.6 Statistical analysis

Data were analyzed using 2 × 2 mixed ANOVA with Group (LKM vs. Control) as the between-subjects factor and Time (Pretest vs. Posttest) as the within-subjects factor for each outcome variable. Bonferroni corrections were applied to adjust for multiple comparisons. Effect sizes were reported using partial eta squared (η^2^) following Cohen’s (26) guidelines, where 0.01, 0.06, and 0.14 correspond to small, medium, and large effects, respectively. Statistical significance was set at *p* < 0.05 (two-tailed). All analyses were conducted using SPSS Version 26.0 (27).

## 4 Results

In this study, a two-arm randomized controlled trial design of 2 (Group: LKM, Control) × 2 (Time: Pre, Post) was adopted, and 2 × 2 mixed ANOVA (2.4) was used to examine the different effects of experimental intervention on IMS, ES, PA, NA, ICS and WWS scores of subjects in the two groups.

Results of Multivariate Tests showed that there was a significant main effect on the Group: *F* (6, 191) = 6.395, *p* < 0.001, η^2^ = 0.167; a significant main effect on Time: *F*(6, 191) = 5.172, *p* < 0.001, η^2^ = 0.140; and a significant interaction effect between Group and Time: *F*(6, 191) = 5.384, *p* < 0.001, η^2^ = 0.145. Table 2 presents the descriptive statistics of all outcome variables across groups and timepoints.

**Table 2:** Descriptive statistics.

Group	Measures	Mean (SD)	
		Pre	Post
LKM (*n* = 50)	IMS	59.60 (14.303)	70.98 (15.663)
	ES	11.06 (2.839)	13.54 (2.950)
	PA	14.42 (4.280)	17.48 (3.721)
	NA	15.62 (4.261)	14.44 (3.170)
	ICS	11.480 (2.801)	13.34 (3.068)
	WWS	16.58 (3.391)	17.87 (4.234)
Control (*n* = 50)	IMS	59.30 (14.701)	59.16 (14.285)
	ES	10.84 (2.411)	10.84 (2.951)
	PA	14.02 (3.000)	14.06 (3.467)
	NA	15.74 (2.709)	15.94 (3.467)
	ICS	11.38 (2.440)	11.52 (3.247)
	WWS	16.58 (3.240)	16.62 (3.434)

IMS, Interpersonal Mindfulness Scale; ES, Empathy Scale; PA, Positive Affect; NA, Negative Affect; ICS, Interdisciplinary Collaboration Scale; WWS, Workplace Well-being Scale.

**Figure 2:**
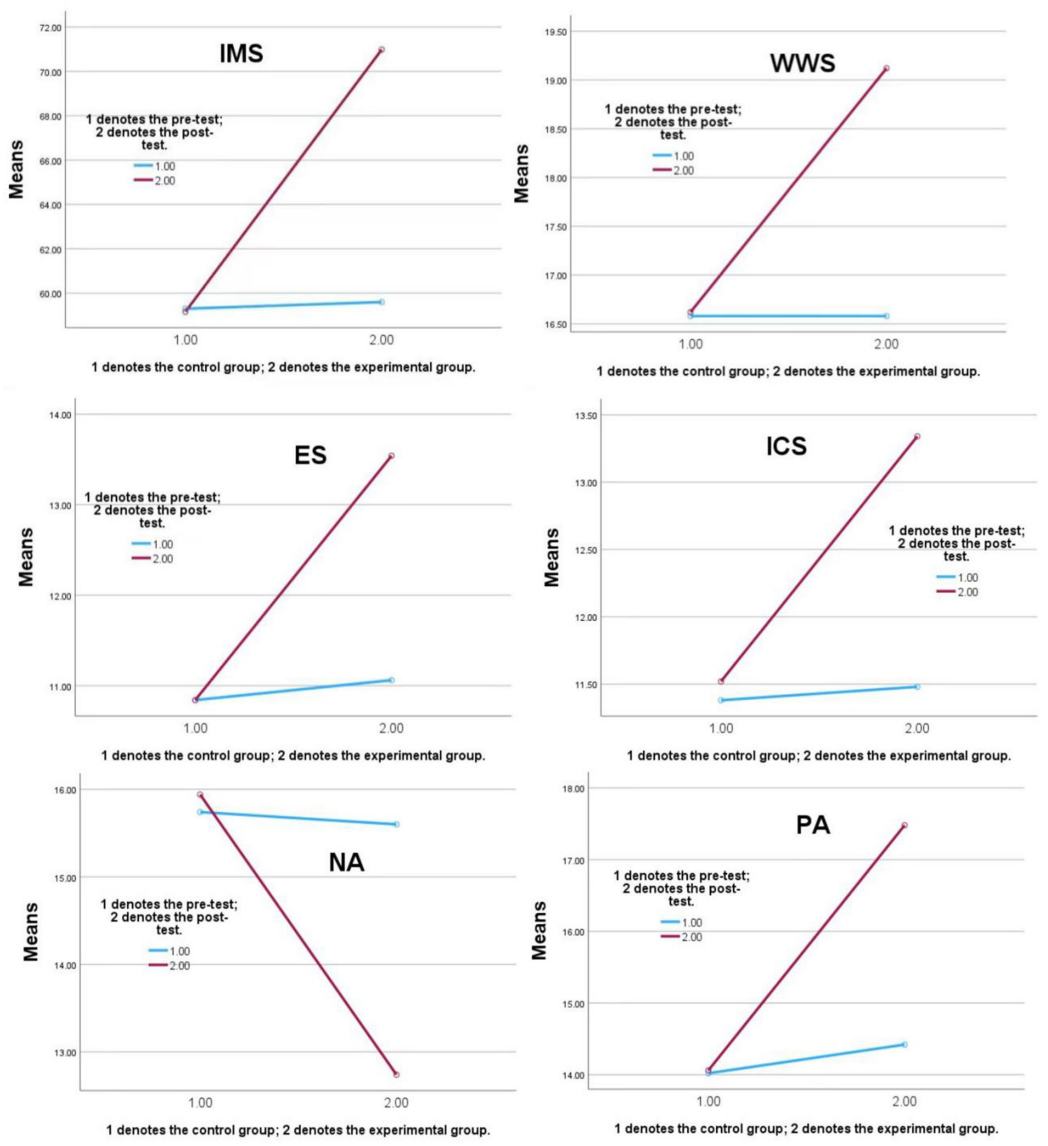
Significant interaction effects between Group and Time. IMS, Interpersonal Mindfulness Scale; ES, Empathy Scale; PA, Positive Affect; NA, Negative Affect; ICS, Interdisciplinary Collaboration Scale; WWS, Workplace Well-being Scale.

The results of Table 3 reveal there significant interaction effects between Group and Time in the fourmeasures (IMS, ES, PA, NA, ICS, and WWS). Figure 2 shows these interaction effects are in more detail.

**Table 3:** Tests of between-subjects effects.

Measures	Effect	*F*	*p*	η^2^
IMS	Group^**^	8.621	0.004	0.042
	Time^**^	7.415	0.007	0.036
	Group × Time^**^	7.789	0.006	0.038
ES	Group^***^	13.630	<0.001	0.065
	Time^**^	9.831	0.002	0.048
	Group × Time^**^	9.831	0.002	0.048
PA	Group^***^	13.727	<0.001	0.065
	Time^**^	9.040	0.003	0.044
	Group × Time^**^	8.580	0.004	0.042
NA	Group^***^	12.115	<0.001	0.058
	Time^**^	7.684	0.006	0.038
	Group × Time^**^	10.169	0.002	0.049
ICS	Group^*^	5.461	0.020	0.027
	Time^**^	9.831	0.002	0.048
	Group × Time^*^	4.832	0.038	0.022
WWS	Group^*^	5.660	0.018	0.028
	Time^*^	6.028	0.015	0.030
	Group × Time^*^	5.660	0.018	0.028

IMS, Interpersonal Mindfulness Scale; ES, Empathy Scale; PA, Positive Affect; NA, Negative Affect; ICS, Interdisciplinary Collaboration Scale; WWS, Workplace Well-being Scale. Group: Between-subjects; Time: Within-subjects; Group × Time: Interaction. *p < 0.05; **p < 0.01; ***p < 0.001.

Among the measured variables, IMS and ES showed the largest statistically significant increases (η^2^ = 0.038 and 0.048, respectively), followed by PA (η^2^ = 0.042) and WWS (η^2^ = 0.028). NA showed a statistically significant reduction (η^2^ = 0.049), while ICS also demonstrated a statistically significant increase (η^2^ = 0.022). Collectively, these results suggest that the short video app–guided Loving-Kindness Meditation (LKM) intervention produced significant improvements across emotional, interpersonal, and occupational dimensions.

All *post hoc* comparisons remained statistically significant after Bonferroni correction, confirming the robustness of these findings. Descriptive statistics for all outcome variables are summarized in Table 2, and the Group × Time interaction effects are visualized in Figure 2. A concise summary of the hypothesis testing outcomes, including corresponding *F*-values, *p*-values, and effect sizes, is provided in Table 4.

**Table 4:** Summary of hypothesis testing outcomes.

Hypothesis	Variable	*F* (1, 96)	*p*	η^2^	Statistical conclusion
H1	IMS	7.789	0.006	0.038	Supported
H2	ES	9.831	0.002	0.048	Supported
H3	ICS	4.832	0.038	0.022	Supported
H4	PA	8.58	0.004	0.042	Supported
	NA	10.169	0.002	0.049	Supported
H5	WWS	5.66	0.018	0.028	Supported

## 5 Discussion

This study demonstrated that a four-week short video–guided Loving-Kindness Meditation (LKM) intervention produced statistically significant improvements in interpersonal mindfulness, empathy, interdisciplinary collaboration, affective balance, and workplace well-being among full-time employees. These findings extend previous mindfulness research by showing that compassion-based meditation, when delivered through short-form digital media, can enhance both emotional and social functioning in contemporary occupational settings. Beyond confirming prior evidence that LKM increases prosocial emotions and decreases negative affect (4, 5, 11), the present results illustrate that even brief, digitally mediated interventions can yield measurable psychological benefits without the logistical burden of traditional in-person programs.

The observed improvements across interpersonal and affective domains can be theoretically understood through the Broaden-and-Build Theory of Positive Emotions (13). LKM systematically cultivates emotions such as warmth, gratitude, and compassion, which broaden individuals’ attentional and cognitive repertoires, promoting more flexible emotion regulation and prosocial orientation (6). Over repeated exposure, this affective expansion fosters enduring psychological resources—empathy, patience, and social trust—that translate into improved collaboration and overall well-being. Prior work has shown that compassion training enhances perspective taking and reduces self-centered bias (28), mechanisms that likely underpin the empathy and teamwork improvements observed in this study. Importantly, participants’ gains in interpersonal mindfulness suggest that compassionate attention enhances awareness of others’ emotional cues while reducing reactivity and defensiveness in daily interactions, consistent with previous findings that compassion-based mindfulness fosters both self-regulation and empathic resonance (29, 30).

From an organizational psychology perspective, the results provide evidence that compassion-based emotional training can influence social and motivational dynamics in the workplace. Employees who engaged in the LKM intervention reported improved interpersonal functioning and affective stability—two predictors of collaborative performance and job satisfaction (2, 31). By encouraging benevolent intentionality (“May you be safe and happy”), LKM may reshape employees’ appraisal of social interactions from threat-based to affiliative frames, increasing psychological safety and openness to cooperation (32). The corresponding reduction in negative affect further supports this mechanism, as emotionally balanced individuals are more resilient to interpersonal stressors and more capable of maintaining constructive engagement (33). Together, these findings suggest that digital LKM interventions can act as preventive strategies for emotional exhaustion and workplace conflict, enhancing collective harmony and well-being.

A notable innovation of this study lies in the use of short video–based delivery for compassion training. Unlike conventional mobile mindfulness applications (e.g., Headspace, Calm) that rely on long audio-guided sessions requiring sustained concentration (34, 35), short video platforms present visually engaging, time-limited segments that better align with users’ everyday digital habits. The cognitive advantage of this format does not stem from the video modality per se but from its brevity, multimodal structure, and emotionally salient presentation, which jointly reduce extraneous cognitive load and enhance attentional capture. According to the Cognitive Theory of Multimedia Learning (36) and Cognitive Load Theory (37), concise multisensory stimuli can distribute information processing across visual and auditory channels, thereby lowering working-memory demands and improving retention. Empirical studies in digital learning similarly show that short, emotionally engaging clips can enhance sustained attention and task adherence in mobile-based interventions (38, 39). This micro-learning structure thus makes short video–guided meditation cognitively accessible and habitually engaging for users.

### 5.1 Theoretical contributions

This study contributes to the theoretical integration of mindfulness and organizational well-being research by empirically validating a compassion-based digital intervention model. First, it supports a multi-mechanism framework in which affective intention (cultivating goodwill), cognitive decentering (reducing self-focus), and relational awareness (attunement to others) jointly mediate the impact of mindfulness on emotional and social outcomes. Second, by demonstrating that micro-dose compassion interventions can yield significant effects, it broadens the conceptual boundary of contemplative science beyond traditional lengthy meditation practices (29). Finally, the study bridges contemplative theory with media psychology, suggesting that engagement design—particularly brevity, interactivity, and personalization—can serve as meaningful vectors for prosocial emotion cultivation in the digital age.

### 5.2 Practical implications

Practically, this study underscores the feasibility of integrating short video–guided Loving-Kindness Meditation (LKM) into organizational wellness and human resource development programs. For example, organizations can embed brief LKM sessions into digital onboarding systems to cultivate empathy and emotional adaptability among new employees (6, 40) or incorporate them into leadership training modules to enhance interpersonal awareness and compassion-based decision-making (9, 41). In hybrid or remote work settings, short video–guided LKM can be delivered through corporate intranet platforms, micro-learning portals, or mobile HR applications, allowing employees to participate flexibly without disrupting workflow (14, 42).

Beyond improving individual well-being, such interventions may help build a culture of compassion and collaboration, supporting prosocial communication and reducing interpersonal tension in high-stress workplaces (20, 43). However, successful integration requires managerial endorsement, organizational readiness, and sustained communication to prevent novelty decay (44). Technical challenges—such as data privacy, content quality control, and measuring engagement fidelity—must also be addressed to ensure long-term success (45).

To overcome some of these barriers, organizations can draw insights from the growing field of digital compassion interventions. Previous compassion-based programs have mainly relied on mobile or web applications using structured audio sessions, reflective exercises, or synchronous group formats (46). While effective, these models often demand significant time commitment and continuous attention, limiting scalability in fast-paced workplaces (47). In contrast, short video–guided LKM adopts a micro-learning design embedded in familiar social-media-style clips, providing emotionally engaging and time-efficient experiences that align with employees’ daily media habits (48). This design lowers cognitive and temporal entry barriers, enhances ecological validity, and enables seamless integration into existing digital ecosystems (13).

Overall, integrating short video–based compassion interventions into organizational health strategies represents a promising yet complex endeavor—one that requires not only technological innovation but also institutional commitment, ongoing evaluation, and a supportive organizational culture to ensure sustainability.

### 5.3 Limitations

Several limitations should be noted. First, the sample size, while sufficient for preliminary testing, limits generalizability across industries and organizational roles, particularly given the variability of workplace cultures and job demands (49). Second, self-report measures may be subject to response bias, social desirability, and common method variance, and they do not directly capture actual behavioral change (50). Third, the duration of the intervention was relatively short, and the long-term maintenance or decay of effects remains unknown, particularly in real-world organizational environments where stressors are ongoing (51). Fourth, the present study employed a no-intervention control group, which may have introduced expectancy and attention biases. Fifth, this study focused on a general employee sample, which limits the generalizability of findings to specific professional groups such as healthcare workers or educators. Finally, although participants were randomly assigned, they were not blinded to condition, which may have introduced expectancy or placebo-like effects that influenced self-reports (52).

### 5.4 Future directions

Future research should explore the longitudinal impact of LKM in professional contexts, including follow-up assessments to examine sustained behavioral and emotional change over time (10). Incorporating objective indicators, such as physiological stress biomarkers (e.g., heart rate variability, cortisol levels), behavioral collaboration metrics, or third-party evaluations, would strengthen the robustness of the findings and reduce reliance on self-reported outcomes (51). Comparative studies between LKM and other mindfulness interventions, such as Mindfulness-Based Stress Reduction (MBSR) and Mindfulness-Based Cognitive Therapy (MBCT), may help clarify the unique contribution of compassion-focused mechanisms in enhancing workplace well-being (11). Future research should include an active control condition (e.g., neutral videos or guided relaxation) to ensure equivalent engagement across groups. Future research should examine whether short video–guided compassion-based interventions yield comparable benefits across high-stress occupational groups such as healthcare professionals and teachers. While these findings are promising, they should be interpreted cautiously given the study’s short duration and specific occupational sample. Future studies should verify their stability and generalizability across different work settings. Finally, investigating moderator variables, including baseline empathy levels, emotional regulation tendencies, or organizational culture characteristics, could inform the design of tailored interventions that maximize impact across different employee subgroups (13).

## 6 Conclusion

In summary, this study demonstrates that a short video–guided Loving-Kindness Meditation (LKM) intervention can serve as a practical and scalable tool for enhancing employees’ interpersonal mindfulness, empathy, collaboration, affect regulation, and workplace well-being. By integrating compassion-based training into an easily accessible mobile format, organizations can provide brief yet effective mental health support without disrupting regular work routines. Such interventions may be particularly valuable in high-stress or communication-intensive occupations, where emotional resilience and prosocial engagement are essential to performance and morale. Nevertheless, future studies should examine the long-term sustainability, optimal delivery frequency, and organizational feasibility of short video–based meditation programs to better understand their role in comprehensive workplace wellness strategies.

## Data Availability

The original contributions presented in the study are included in the article/supplementary material, further inquiries can be directed to the corresponding author.
